# Gene expression profiling by RNA-sequencing reveals regulators of intramuscular fat in Black Slavonian pigs

**DOI:** 10.1038/s41598-026-52510-x

**Published:** 2026-05-20

**Authors:** Boris Lukic, Goran Lipavić, Mateja Bulaić, Curik Ino, Žarko Radišić, Ras Lužaić, Nikola Raguž

**Affiliations:** 1 Department for Animal Production and Biotechnology, Faculty of Agrobiotechnical Sciences Osijek, J.J. Strossmayer University of Osijek, Osijek, Croatia; 2 Central Finance and Contracting Agency, Zagreb, Croatia; 3 Inspecto Ltd., Osijek, Croatia; 4 Department of Animal Science, Faculty of Agriculture, University of Zagreb, Zagreb, Croatia; 5 Hungarian University of Agriculture and Life Sciences - Institute of Animal Sciences (MATE), Kaposvár, Hungary

**Keywords:** Pig, Gene expression, RNA-sequencing, Intramuscular fat IMF, Genetics, Molecular biology

## Abstract

Intramuscular fat (IMF) plays an important role in determining meat quality traits such as flavor, tenderness, and juiciness. While numerous studies have investigated the genetic basis of IMF in commercial pig breeds, data on local breeds remain rather limited. In this study, we used RNA-sequencing to characterize the transcriptomic differences between high-IMF and low-IMF Black Slavonian pigs, a native Croatian breed known for superior meat quality. Muscle samples (*Longissimus thoracis et lumborum*) from 14 pigs with divergent IMF levels were collected shortly after slaughter, preserved in liquid nitrogen, and stored at − 80 °C until RNA extraction. Intramuscular fat content was determined from the same muscle 24 h post mortem using the Soxhlet extraction method (ISO 1443:1973). These samples were then analyzed to identify differentially expressed genes (DEGs) and enriched pathways. A total of 519 genes were differentially expressed (*p* ≤ 0.05), with 457 remaining significant after false discovery rate correction. The high-IMF group exhibited upregulation of genes associated with lipid metabolism (e.g., SCD, ADIPOQ, CIDEC, FABP4), PPAR signaling, and adipogenesis, while genes linked to muscle structure and oxidative metabolism were downregulated. Functional enrichment and gene set enrichment analyses highlighted coordinated regulation of pathways related to fatty acid biosynthesis, extracellular matrix (ECM) remodelling, angiogenesis, and Notch signaling. Notably, several ECM-related genes (LAMA1, TIMP4) and angiogenic factors (FGF2, NRP1) were significantly upregulated, suggesting that adipocyte expansion in muscle requires parallel vascular and structural adaptations. Importantly, several of the top 30 DEGs, including EHD2, NRG4, UTRN, FLNA, and HMCN1, represent novel candidate genes not previously linked to IMF in pigs, pointing to potential breed-specific mechanisms. These genes are associated with membrane trafficking, paracrine signalling, cytoskeletal re-modelling, and ECM dynamics. Our findings contribute new molecular insights into IMF regulation in local pig breeds and provide a foundation for developing targeted breeding strategies to improve pork quality through intramuscular fat enhancement.

**Supplementary Information:**

The online version contains supplementary material available at 10.1038/s41598-026-52510-x.

## Introduction

In the last decades, trends in genetic evaluation of pigs have changed from growth and muscularity traits to traits like feed efficiency (e.g. residual feed intake), and especially to meat quality traits^1^. Intramuscular fat content (IMF) is considered as one of the most important indicators of meat quality, due to its positive effects on flavour or juiciness of meat^2^, trait especially important for the nowadays consumers. Generally, in pigs, IMF refers to the lipid content found within the muscle tissues, typically of loin (*Longissimus muscle*) or ham (*Musculus gluteus*). It is distributed among the muscle fibres and is influenced by genetic, nutritional, and other environmental factors. Fat cells or adipocytes, form through adipogenesis, a process closely tied with the formation of two other main tissues, muscle and connective, which form through a processes of myogenesis and fibrogenesis, respectively^3^. IMF depends on the number and size of the adipocytes, but also on the level of fat metabolism e.g. fat synthesis (lipogenesis) and fat breakdown (lipolysis). The main function of fat tissue is energy storage, but it also plays important role in endocrine system, as it serves for the hormone production^4^.

In the context of breeding, IMF is a complex and highly heritable trait with h^2^ estimates of 0.5–0.6^5,6^, however, depending on the breed and method of IMF detection, h^2^ estimates show wide range as some studies^7,8^ reported quite low (~ 0,2) and high (~ 0.8) h^2^ values^6^. This is understandable due to the previously described complexity of biological background. It is difficult to measure IMF on live animal, since reliable levels are available only after slaughter^1^, and consequently to genetically evaluate animals based on the IMF values. Also, for several decades increasing the carcass leanness was primary objective in commercial pig breeding programs, and as consequence, IMF was dramatically decreased due to the negative genetic correlations between the carcass leanness and IMF^9^. Nevertheless, the utilization of IMF content in breeding programs is a highly complex task, as its polygenic nature is clearly illustrated with nearly 900 QTL-s associated with IMF registered in the Pig QTL database (www.animalgenome.org/).

Local pig breeds throughout the world represent an important reservoir of genetic diversity, with a large number of allelic variants affecting IMF identified mainly by GWAS^10–14^. Black Slavonian (BS) pig is a local autochthonous breed in Croatia, systematically developed in the nineteenth century^15^. This breed exhibits lower growth and reproduction performance, however, it simultaneously shows notably higher meat quality compared to hybrid pigs^16^ and particularly values of IMF higher than 10% (in publish). Therefore, this breed represents suitable animal to analyse genes affecting IMF.

Generally, GWAS studies on both commercial and local breeds detected numerous genomic locations on almost every porcine chromosome^8,10–13,17–20^, with several confirmed between studies. However, GWAS approach essentially identifies genomic regions related to IMF, without the knowledge about the biological and functional significance or exact causal variant^21^. With the advent of Next generation sequencing technologies, RNA-sequencing (RNA-Seq) method overcomes these limitations but also provides deeper and more accurate understanding of genomics and transcriptomics research in livestock species^22^. RNA-Seq allows direct insight into gene activity at the transcriptional level, enabling researchers to identify genes that are differentially expressed between animals with high and low IMF content. This functional perspective is crucial for deciphering the complex regulatory mechanisms underlying fat deposition in muscle tissue. Moreover, RNA-Seq captures novel transcripts, isoforms, and non-coding RNAs that may be overlooked by traditional genotyping arrays but can still play important roles in adipogenesis. As such, transcriptomic profiling complements genomic approaches and is particularly valuable for local breeds like the Black Slavonian pig, where reference panels and SNP coverage may be limited. Using RNA-Seq, it is possible to analyse gene expression and accurately quantify entire transcripts expressed in any tissue. Therefore, the aim of this study was to analyse transcriptomic profiles of *Musculus thoracis muscle* of Black Slavonian pig with divergent IMF phenotypes and to identify gene networks and pathways in which the differentially expressed genes are involved (DEGs). In this way, we can identify candidate genes affecting IMF in pigs and use this information in future breeding program.

## Results

The results on main carcass traits of pigs used in this study are shown in Table 1. Hot carcass weight and muscle thickness were significantly higher in the HIMF group compared to the LIMF group (*p* = 0.01). In contrast, backfat thickness and dressing percentage did not differ significantly between groups. As expected from the experimental design, the HIMF group showed an average IMF content of 8.20% (min = 5.80%, max = 10.50%), while the LIMF group showed an average of 2.30% (min = 1.60%, max = 3.30%). To provide a comprehensive overview, Table 1 presents summary statistics both for the entire group of animals included in the study (n = 80) and separately for the HIMF and LIMF subsets (n = 7 each), which were selected for RNA-sequencing analysis.

**Table 1:** Main carcass traits of pigs used in this study.

Groups	ALL	HIMF	LIMF
Hot carcass weight (kg)	118.35 ± 15.79	128.10 ± 12.16	109.70 ± 20.44
Dressing percentage (%)	83.54 ± 1.61	84.08 ± 1.48	83.24 ± 1.61
Muscle thickness, M (mm)	64.15 ± 6.82	63.80 ± 7.07	50.20 ± 12.04
Fat thickness, F (mm)	57.01 ± 10.80	64.70 ± 6.52	64.80 ± 7.52
IMF	4.46 ± 2.04	8.20 ± 1.44	2.30 ± 0.58

Main phenotypic data of analysed animals. The results are shown as mean ± SD.

IMF (Intramuscular fat); ALL (all animals in analysis; N = 80); HIMF (high intramuscular fat; N = 7); LIMF (low intramuscular fat; N = 7).

### Statistical summary of the RNA-seq analysis

**Fig. 1:**
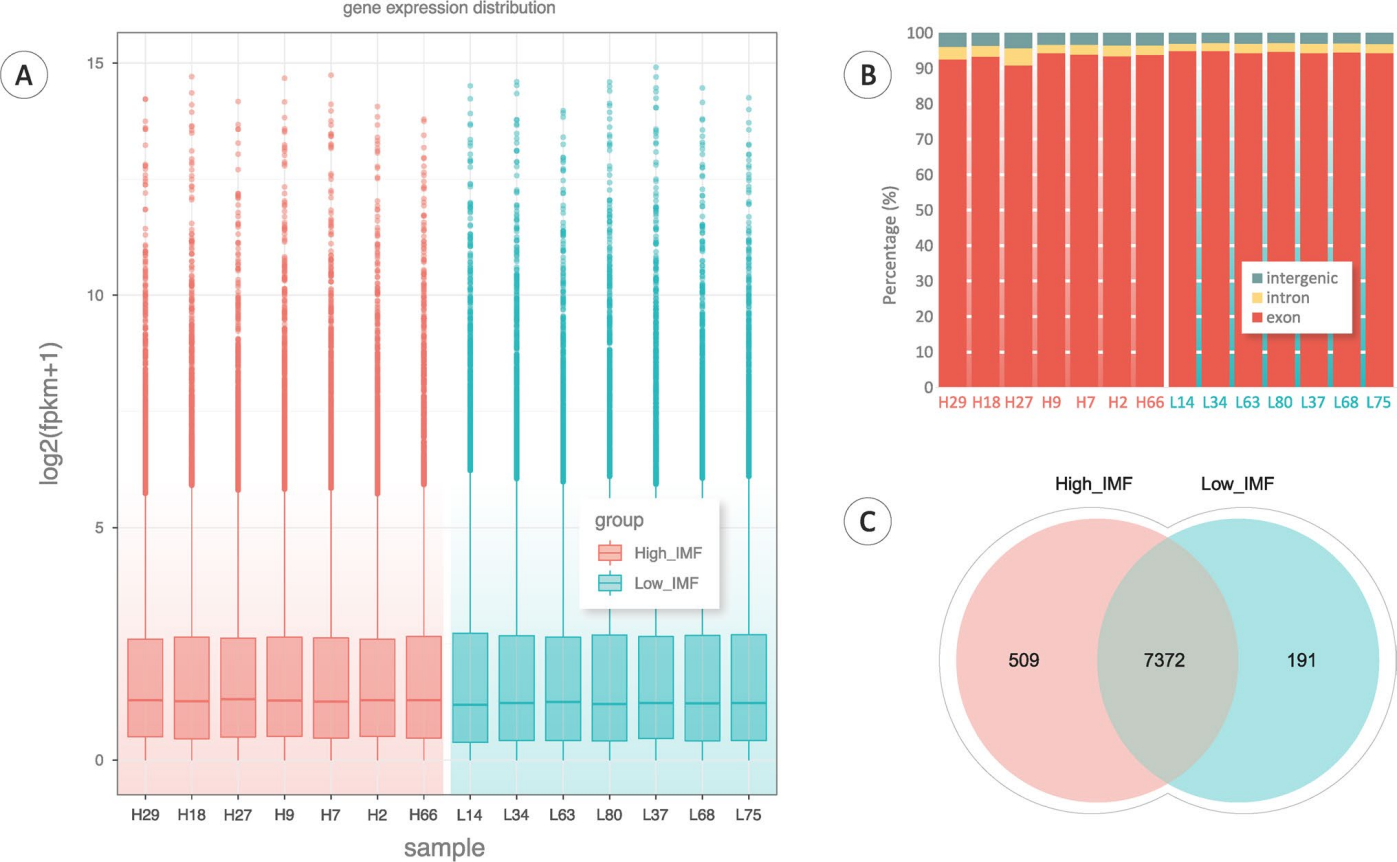
Overview of gene expression distribution and transcriptomic annotation. (**A**) Boxplot showing the distribution of gene expression values (log_2_ FPKM) across all 14 samples. Each box represents one sample from either the high-IMF (HIMF) in red or low-IMF (LIMF) group in green color. (**B**) Bar plot displaying the genomic distribution of mapped reads across intergenic, intronic, and exonic regions, reflecting transcriptomic annotation and read classification. (**C**) Venn diagram illustrating the overlap of expressed genes between the HIMF and LIMF groups, showing shared and group-specific transcriptomic profiles.

**Fig. 2:**
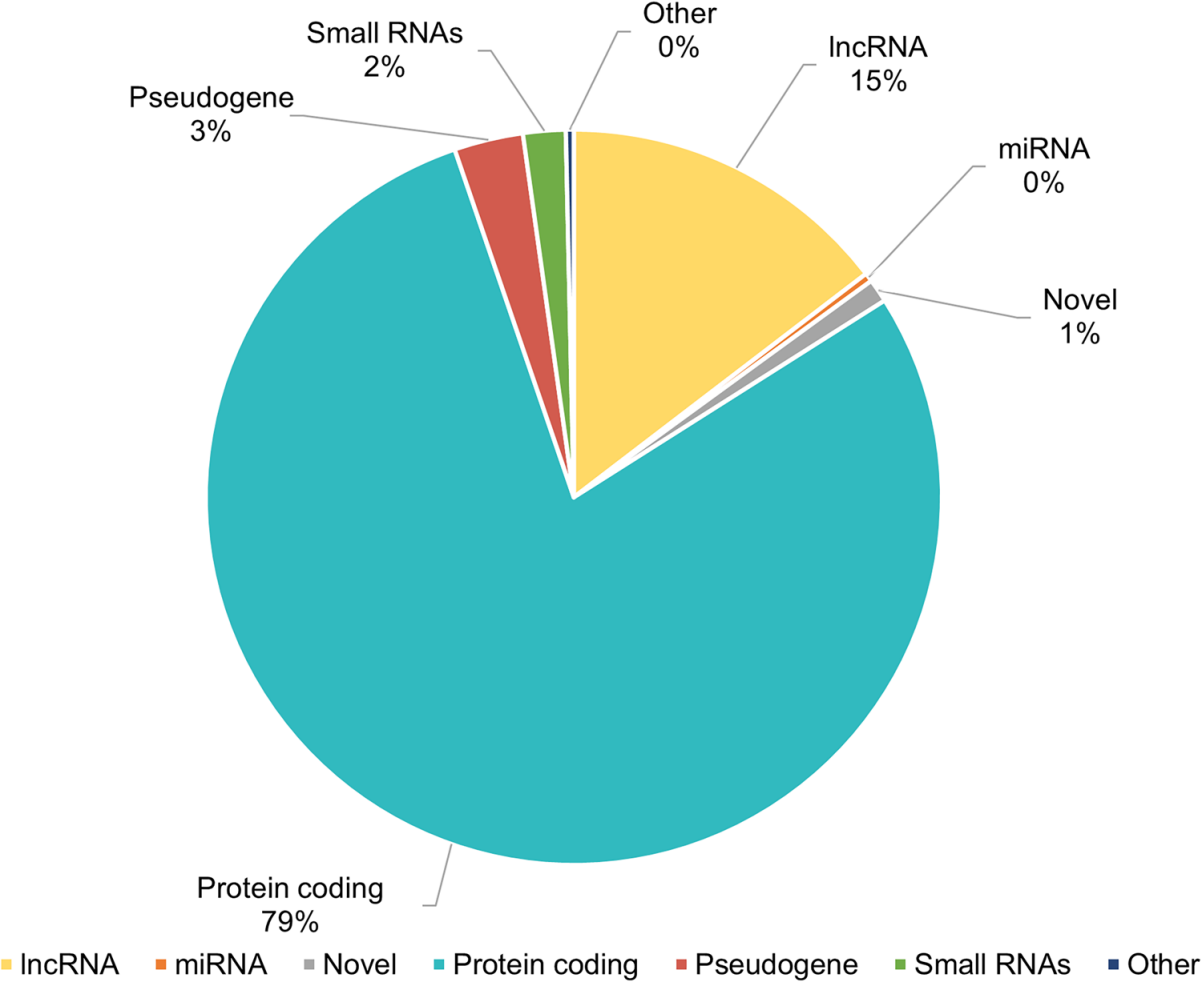
Classification of expressed gene biotypes in porcine muscle transcriptome. Pie chart showing the distribution of gene biotypes based on Ensembl annotation of reads detected in poly-A-enriched RNA-seq data from porcine muscle. As expected, the majority of expressed transcripts were protein-coding (79%), followed by annotated long non-coding RNAs (lncRNAs; 15%), pseudogenes (3%), small RNAs (2%), and a small proportion of novel genes (1%). Given the polyadenylation-based mRNA enrichment method, these proportions reflect only poly-A-containing transcripts and may underrepresent non-polyadenylated RNAs.

**Fig. 3:**
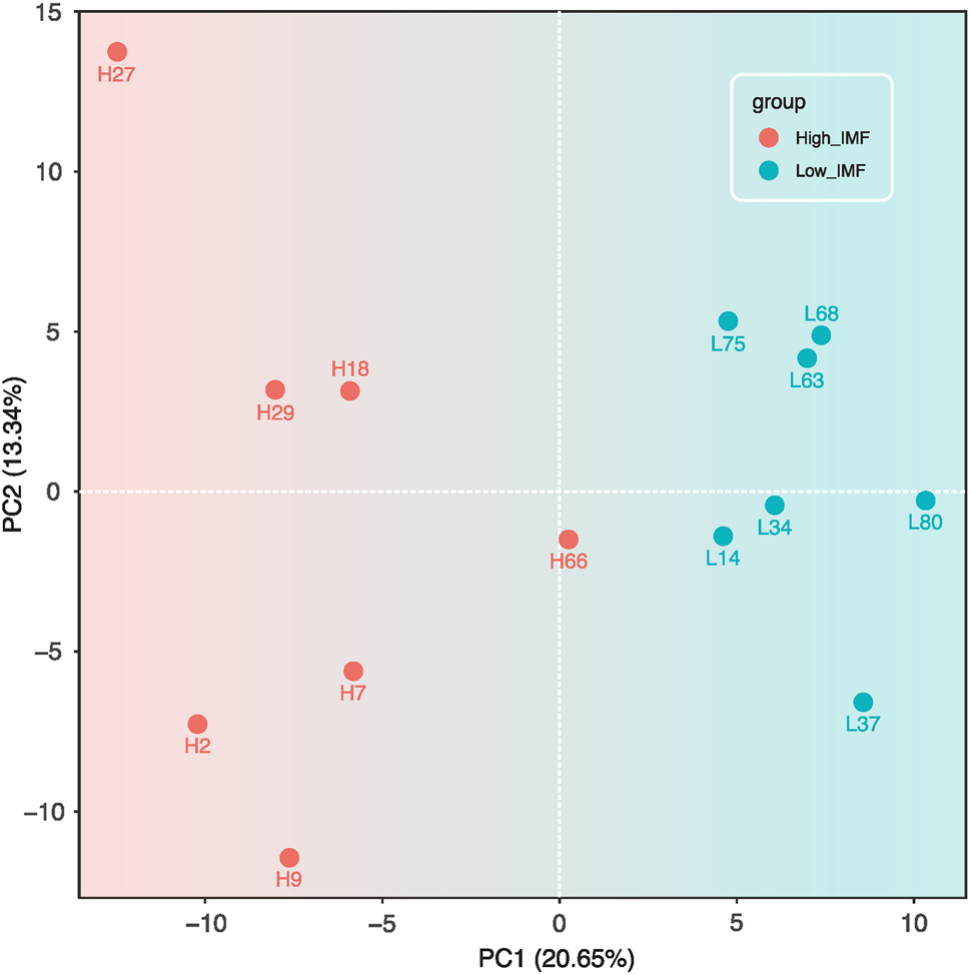
Principal Component Analysis (PCA) of gene expression profiles. PCA plot based on FPKM-normalized expression values for all detected genes across 14 muscle samples. The first two principal components (PC1 and PC2) are shown, capturing the major variation in the transcriptomic profiles. Samples are clearly separated according to intramuscular fat phenotype (HIMF vs. LIMF), indicating distinct gene expression signatures between high and low IMF groups.

RNA in this study was isolated from muscle tissues obtained from two divergent group of totally 14 pigs (7 LIMF and 7 HIMF) and subjected to RNA-seq analysis. The NovaSeq 6000 sequencing platform produced an average of 6.62 Gb of raw reads per sample, with an average of 98.05% clean reads per sample, yielding 6.49 Gb. Average percentage of total reads aligned to the genome of all samples was 95.18%, while the average percentage of the uniquely mapped reads to the current version of the pig genome (*Sscrofa 11.1*) was 90.85% (Supplementary Information Table S1). To confirm RNA-seq data quality, we assessed the genomic distribution of mapped reads, with the majority (93.79%) aligning to exons, indicating successful mRNA enrichment and supporting the reliability of subsequent differential expression analysis (Fig. 1B; Supplementary Information Table S2). Following quantification of gene expression using FPKM, we analysed the expression patterns of genes across all samples (more details in Supplementary Information Table S3). A total of 20,677 genes were identified in the LTL muscle tissue of Black Slavonian pigs, with 7372 genes expressed across the two divergent groups (Fig. 1C). As poly-T oligo enrichment was used for library preparation, the sequencing primarily captured polyadenylated RNAs, leading to the dominance of protein-coding genes and certain long non-coding RNAs among the detected biotypes. Most of the genes detected were protein coding genes (79%), followed by long non-coding RNAs (15%), pseudogenes (3%), small RNAs 2% (scaRNA, snoRNA, snRNA, rRNA), novel genes (1%) and other below 1% (Fig. 2). To further assess the differences between the divergent groups, PCA analysis was conducted (Fig. 3), with the first two components accounting for 34% of the total variation. The separation of samples between the groups was clear and evident; however, greater dispersion was observed within the HIMF group, particularly along the second component.

### Analysis of differentially expressed genes

**Fig. 4:**
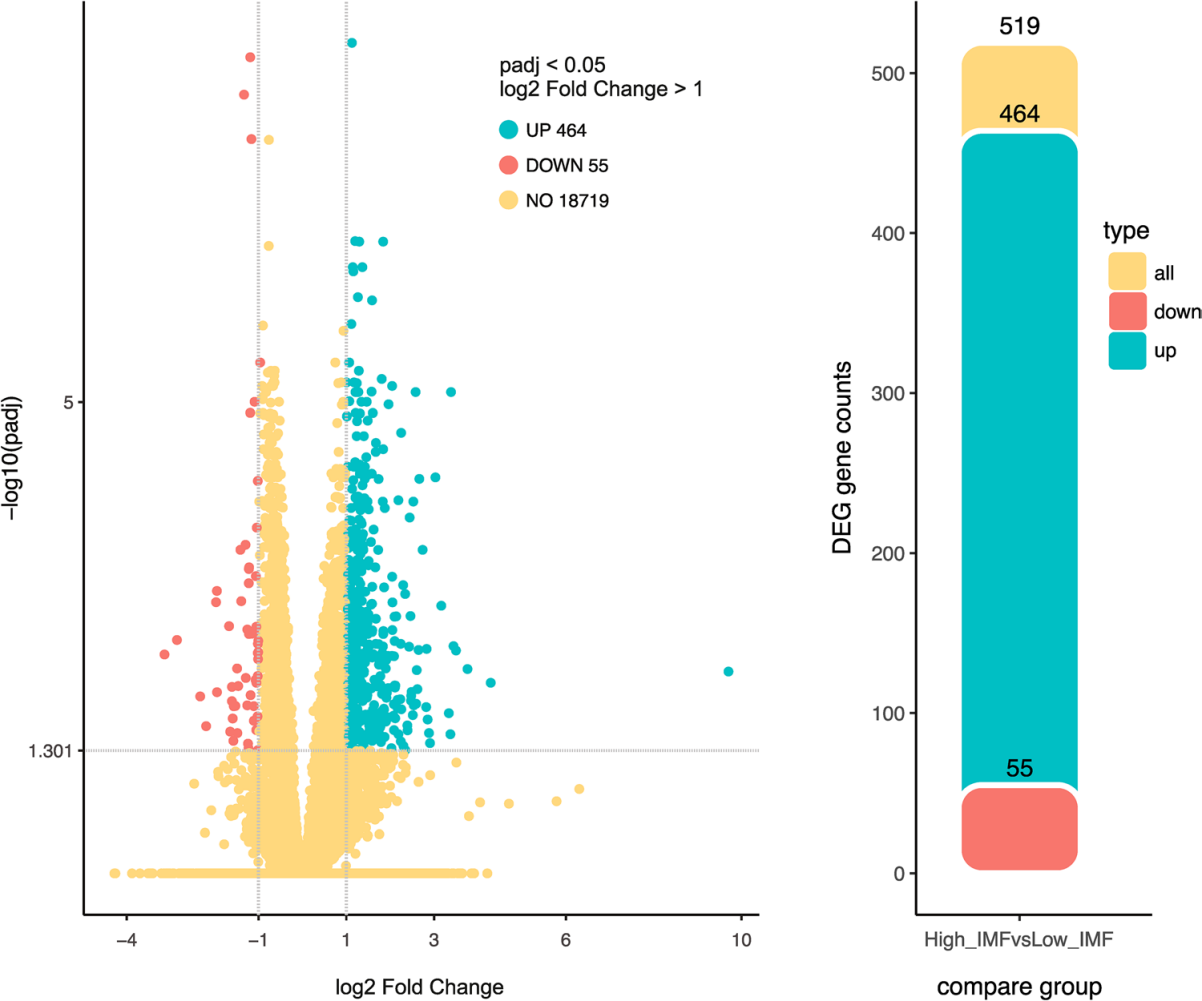
Volcano plot of differentially expressed genes (DEGs) between HIMF and LIMF groups. The x-axis represents log_2_ fold change (log_2_FC), while the y-axis shows –log₁₀ adjusted *p*-values (padj). Each point represents a gene, with significantly upregulated genes (padj ≤ 0.05 and log_2_FC > 1) shown in green, significantly downregulated genes (padj ≤ 0.05 and log_2_FC < –1) in red, and non-significant genes in yellow; all based on FPKM-normalized gene expression values.

A total of 519 genes were differentially expressed between the high intramuscular fat (HIMF) and low intramuscular fat (LIMF) groups at *p* ≤ 0.05 (Fig. 4) and remained significant after false discovery rate correction (P adj ≤ 0.05). List of all differentially expressed genes and statistics are available in Supplementary Information file 4 (Table S4). Among totally differentially expressed genes, 464 genes were upregulated and 55 were downregulated in the LIMF and HIMF groups. The volcano plot illustrates the distribution of differential expression, highlighting the clear separation of highly significant DEGs from the bulk of non-significant genes.

**Fig. 5:**
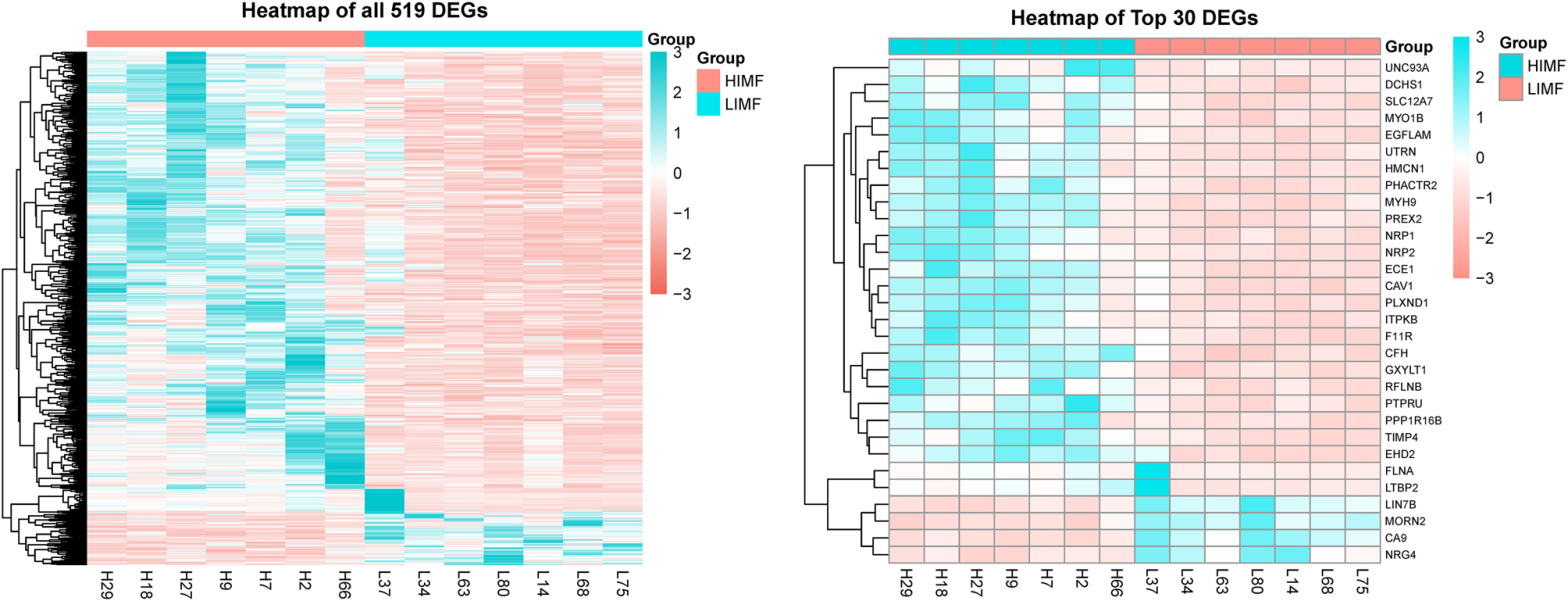
Heatmaps of differential gene expression between HIMF and LIMF groups in Black Slavonian pigs. (**A**) Hierarchical clustering heatmap based on FPKM values of all 519 differentially expressed genes (DEGs) (adjusted *p*-value ≤ 0.05) in LTL muscle of pigs with divergent IMF content. Expression values are scaled as Z-scores, and clustering shows distinct transcriptional profiles between HIMF and LIMF groups. (**B**) Heatmap of the top 30 most significant DEGs ranked by adjusted *p*-value, using FPKM expression values. Genes displayed show pronounced expression divergence between HIMF and LIMF pigs and are associated with pathways including lipid metabolism, extracellular matrix remodelling, and vascular development.

To visualise overall transcriptional differences between the high and low IMF groups, we generated a hierarchical clustering heatmap of all 519 differentially expressed genes (padj ≤ 0.05) across the 14 samples. The heatmap revealed a clear separation between HIMF and LIMF samples, with consistent expression patterns clustering by phenotype (Fig. 5a and b). Although individual gene labels were omitted due to space constraints, the global heatmap provides a visual confirmation of strong transcriptomic divergence between groups.

We first examined the top 100 DEGs to identify genes associated with key biological pathways, including vascular regulation (e.g. ROBO4, BDKRB2, PECAM1, FLT1), extracellular matrix organisation (e.g. ITGA6, JAM2, MATN2), and developmental or signalling processes (e.g. SHROOM4, MEOX1, SRGAP2). To highlight the most relevant gene-level changes, a heatmap was subsequently plotted displaying the top 30 DEGs ranked by adjusted *p*-value (Fig. 5B). This focused visualisation illustrates group-specific expression patterns and includes several candidate genes functionally related to intramuscular fat biology.

### Functional enrichment analysis of DEGs

**Fig. 6:**
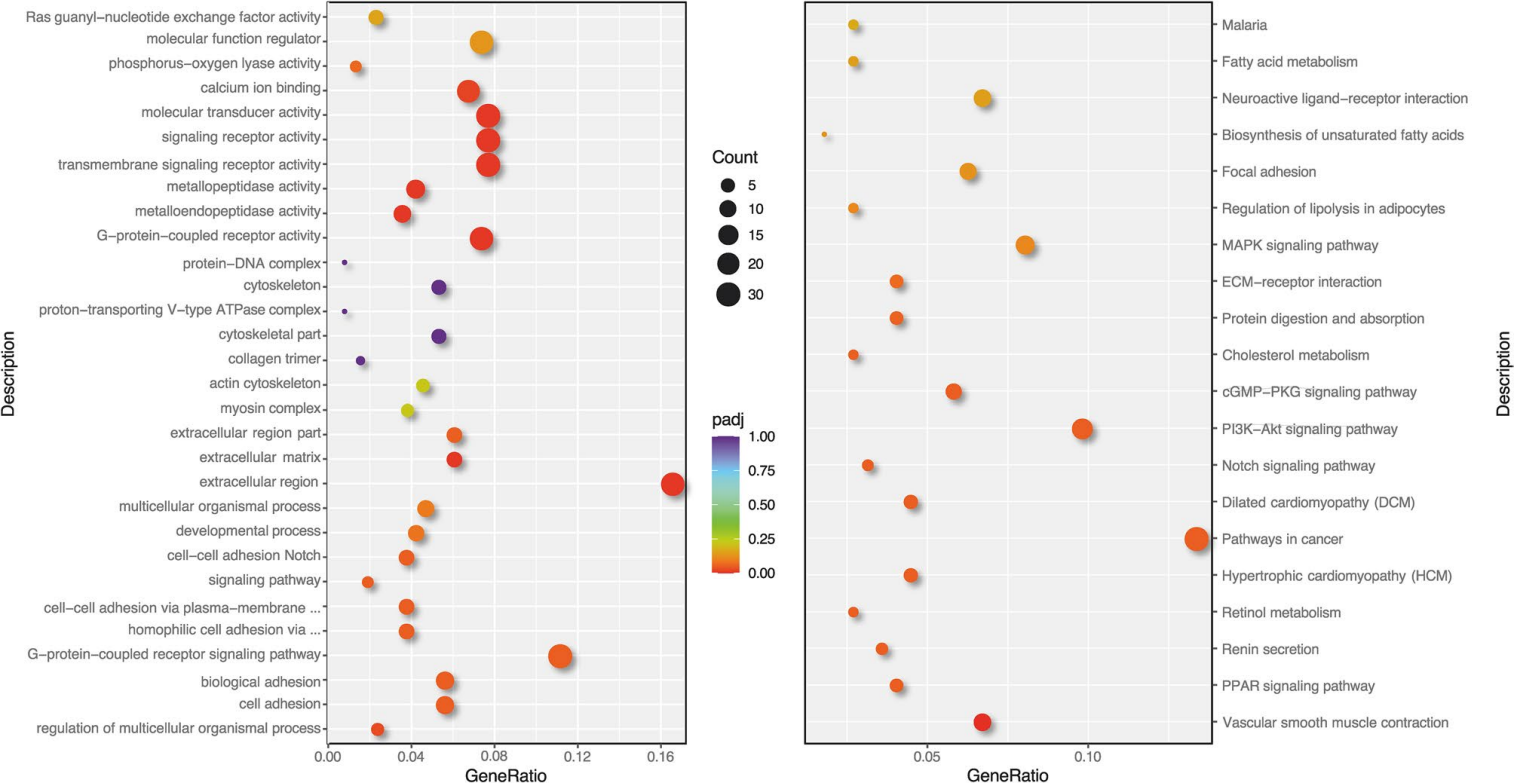
Significantly enriched GO terms (**A**) and KEGG pathways (**B**). (**A**) Gene Ontology (GO) enrichment analysis showing the top significantly enriched biological process terms (padj ≤ 0.05) among differentially expressed genes (DEGs), highlighting processes related to lipid metabolism, extracellular matrix organization, and vascular development. (**B**) Kyoto Encyclopedia of Genes and Genomes (KEGG) pathway enrichment analysis of DEGs, identifying significantly enriched pathways including PPAR signaling, ECM-receptor interaction, and regulation of lipolysis in adipocytes. Color scale represents the adjusted *p*-values, and dot size indicates the number of genes associated with each term or pathway.

Gene Ontology (GO) enrichment analysis was performed on the subset of 218 differentially expressed genes (DEGs) that were successfully annotated to GO terms (Table 2 and Fig. 6A), out of the totally identified DEGs at P adj ≤ 0.05. A total of 10 significantly enriched GO categories (all available in Supplementary Information Table S5) were identified (padj ≤ 0.05), the majority of which fell under the Molecular Function (MF) domain (Fig. 6A). The functional enrichment analysis was conducted using the clusterProfiler R package, which adjusts for gene length bias and controls false discovery rates, ensuring robust statistical inference. GO terms and KEGG pathways were interpreted to identify biological processes and molecular functions relevant to intramuscular fat regulation.

**Table 2:** Significantly enriched GO categories (padj ≤ 0.05) identified in RNA-seq comparison of HIMF and LIMF groups.

GO category	GO term description	P adj	Gene count	Key genes
MF	G-protein-coupled receptor activity	0.0067	23	ADGRL4, ADGRF5, BDKRB2
CC	Extracellular region	0.0072	22	ADAMTS20, IGFBP2, THBS4
CC	Extracellular matrix	0.0072	8	ADAMTS20, MMP28, COL12A1
MF	Metallopeptidase activity / metalloendopeptidase activity	0.0080	13	MMP28, ADAMTS5, ENPP2
MF	Signaling receptor activity / molecular transducer activity	0.0080	24	ADGRF5, CALCRL, OPRL1
MF	Calcium ion binding	0.0188	21	NOTCH4, THBS4, LTBP2
BP	Regulation of multicellular organismal process	0.0296	5	STING1, LAMA3, EDN1

Among the most significantly enriched terms were G-protein-coupled receptor (GPCR) activity (GO:0004930, padj = 0.0067), represented by 23 of 218 annotated DEGs (10.6%), including genes such as ADGRF5, BDKRB2, CALCRL, OPRL1, and ADGRL4. These genes are involved in neuropeptide and hormone signalling, vascular tone regulation, and sensory transduction, suggesting enhanced receptor-mediated intercellular communication in animals with higher IMF content.

Enrichment was also observed in GO categories related to the extracellular matrix (ECM) and extracellular region (GO:0031012, GO:0005576, both padj = 0.0072), supported by 8 to 22 DEGs, including ADAMTS20, MMP28, COL12A1, and THBS4. These genes regulate ECM remodelling, tissue integrity, and cell–matrix interactions, potentially reflecting microstructural adaptations in muscle that facilitate intramuscular fat deposition. Similarly, metallopeptidase and metalloendopeptidase activity (GO:0008237, GO:0004222) were significantly enriched (padj = 0.0081), represented by 13 of 218 DEGs (6.0%), including matrix-modifying enzymes such as ADAMTS5, MMP19, and ENPP2.

Additional enrichment was found for signalling receptor activity and molecular transducer activity (GO:0004888, GO:0038023, P adj = 0.0081), each including 24 DEGs (11.0%), further supporting the involvement of membrane signalling processes in IMF-related transcriptomic profiles. The GO term calcium ion binding (GO:0005509, padj = 0.0188) encompassed 21 DEGs, such as NOTCH4, LTBP2, THBS4, and HMCN1, which participate in developmental signalling and ECM anchoring. The only significantly enriched Biological Process term was regulation of multicellular organismal process (GO:0051239, padj = 0.0296), grouping genes involved in morphogenetic and structural regulation, including LAMA1, EDN1, and STING1. These enriched terms were selected for biological interpretation because they reflect known and novel physiological processes that may underlie intramuscular fat deposition, beyond core lipid metabolism.

Overall, the enriched GO terms point to transcriptomic shifts that extend beyond lipid metabolism, implicating receptor signalling, extracellular matrix remodelling, and tissue structural organisation as complementary processes in the regulation of intramuscular fat content in the LTL muscle of Black Slavonian pigs.

KEGG pathway enrichment analysis was performed on the subset of 226 differentially expressed genes annotated to KEGG terms (Fig. 6B). One pathway reached statistical significance after multiple testing correction (padj ≤ 0.05): vascular smooth muscle contraction (ssc04270, padj = 0.0016). This pathway was represented by 15 of 226 annotated DEGs (6.6%), including genes such as AGT, NPR1, ADCY5, MYLK, ACTA2, and CALCRL, which are key regulators of vascular tone, smooth muscle contraction, and intracellular calcium signalling. Their coordinated upregulation in the HIMF group suggests that vascular remodelling and smooth muscle contractility may be altered in muscle tissue with higher levels of intramuscular fat.

Although additional pathways, including PPAR signalling, renin secretion, and retinol metabolism, showed nominal enrichment (padj ≈ 0.051), they did not pass the significance threshold after correction. Nevertheless, they featured biologically relevant genes such as ADIPOQ, SCD, PPARG, and APOA1, which are involved in lipid metabolism, adipocyte function, and energy regulation.

These KEGG results provide a complementary perspective to the GO enrichment analysis, which highlighted terms related to receptor signalling, extracellular matrix organisation, and metallopeptidase activity. Together, the two analyses suggest that the transcriptomic differences between HIMF and LIMF pigs are not only driven by lipid metabolic processes but also involve broader physiological changes, particularly those related to vascular signalling, contractile function, and structural remodelling of the muscle microenvironment (Table 3).

**Table 3:** Significantly enriched KEGG pathways (padj ≤ 0.05) identified in RNA-seq comparison of high and low IMF groups.

Pathway	P adj	Gene count	Key genes
Vascular smooth muscle contraction	0.0016*	15	AGT, NPR1, ADCY5, MYLK, ACTA2, CALCRL
PPAR signaling pathway	0.0510	9	FABP4, SCD, PLIN5, ADIPOQ, APOA1, PPARG
Renin secretion	0.0510	8	AGT, ADCY5, GUCY1A1, EDN1, EDNRA
Retinol metabolism	0.0510	6	RETSAT, SDR16C5, AOX1
Hypertrophic cardiomyopathy (HCM)	0.0510	10	ACTC1, ITGA6, EDN1, LAMA1

* passes the ≤ 0.05 threshold.

**Fig. 7:**
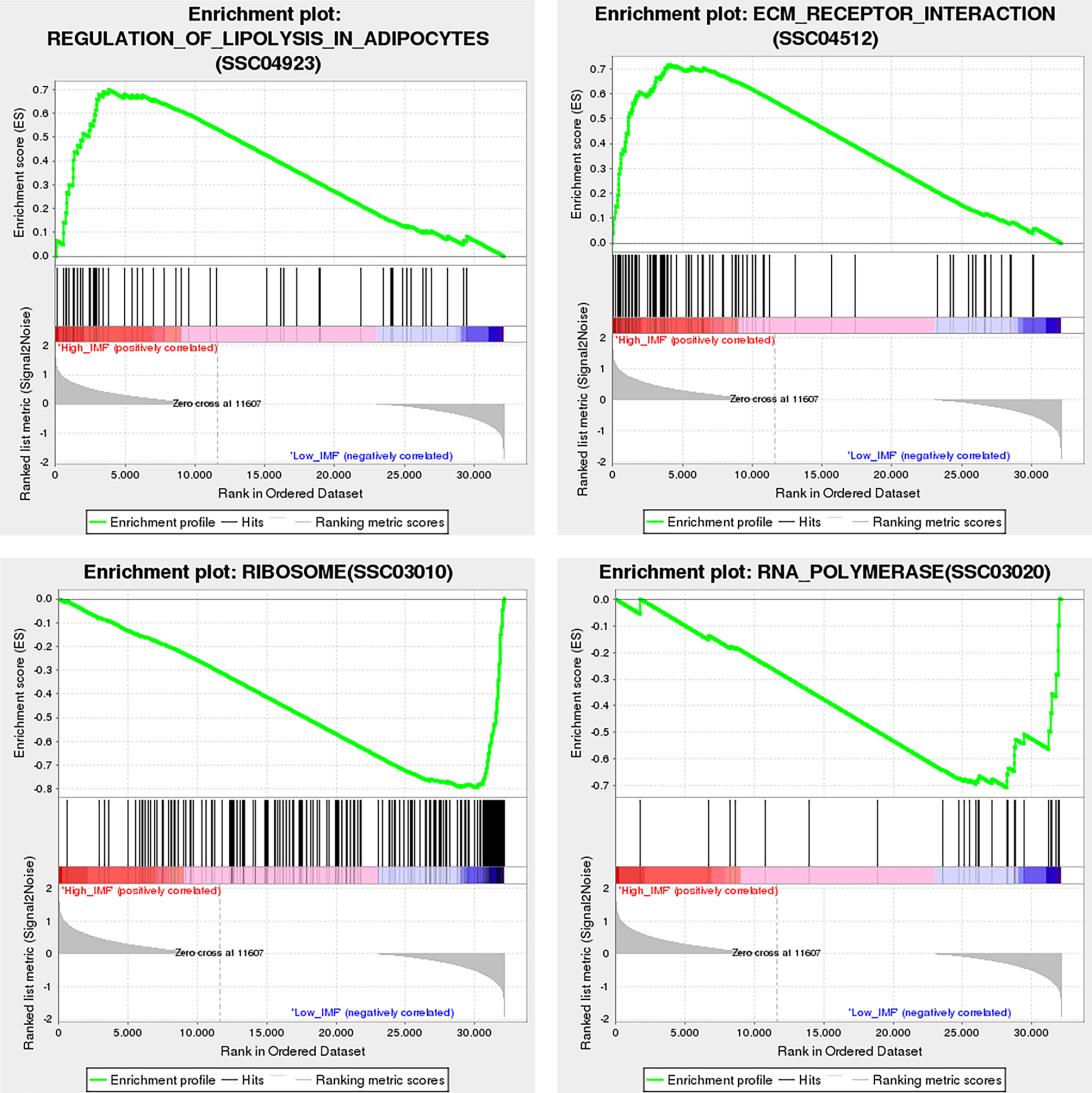
GSEA enrichment plots for top pathways in HIMF and LIMF groups. (**A**) Regulation of lipolysis in adipocytes and (**B**) ECM–receptor interaction were enriched in HIMF. (**C**) Ribosome and (**D**) RNA polymerase were enriched in LIMF. Enrichment score (y-axis) represents the running sum statistic as genes are ranked from most upregulated in HIMF to most upregulated in LIMF. Vertical ticks indicate positions of pathway genes.

Gene Set Enrichment Analysis (GSEA) was performed to detect coordinated shifts in biological pathways between HIMF and LIMF groups using a ranked list of all expressed genes. A total of 315 gene sets were analysed, of which 72 were significantly enriched in the HIMF group and 6 in the LIMF group at FDR < 25%. In the HIMF group, the most significantly enriched pathways included regulation of lipolysis in adipocytes, ECM–receptor interaction, and Notch signalling. These pathways align with biological processes identified in GO and KEGG analysis and suggest coordinated upregulation of adipocyte metabolic activity, extracellular matrix remodelling, and developmental signalling in pigs with higher intramuscular fat content (Fig. 7A and B). In contrast, GSEA for the LIMF group revealed enrichment of core transcriptional and translational processes, including the ribosome, RNA polymerase, and spliceosome pathways (Fig. 7C and D). These pathways were enriched due to consistent upregulation of genes involved in protein synthesis, potentially reflecting a more metabolically active lean muscle phenotype. The divergence between the HIMF and LIMF GSEA profiles confirms that transcriptomic differences extend beyond individual DEGs and encompass broader regulatory programs involving tissue development, structural remodelling, and metabolic capacity.

### Protein–protein interaction (PPI) network analysis

To further explore the functional associations among the differentially expressed genes (DEGs), a protein–protein interaction (PPI) network was constructed based on the STRING database using a minimum interaction confidence score of 700. Out of the 519 DEGs identified, 186 were successfully mapped and retained for network construction after excluding nodes without matched Ensembl gene IDs or without interaction partners at the selected confidence threshold.

The resulting network comprised 186 nodes and 343 interaction edges, illustrating a dense interaction landscape among genes potentially involved in IMF deposition. Each node in the network represents a DEG, and edge thickness reflects the STRING-derived interaction confidence. Node size corresponds to degree centrality, indicating the number of direct interactions for each gene. Several hub genes with high connectivity were observed, including SCD, FASN, ELOVL6, and PLIN1, all of which are well-established regulators of lipid metabolism and adipogenesis.

**Fig. 8:**
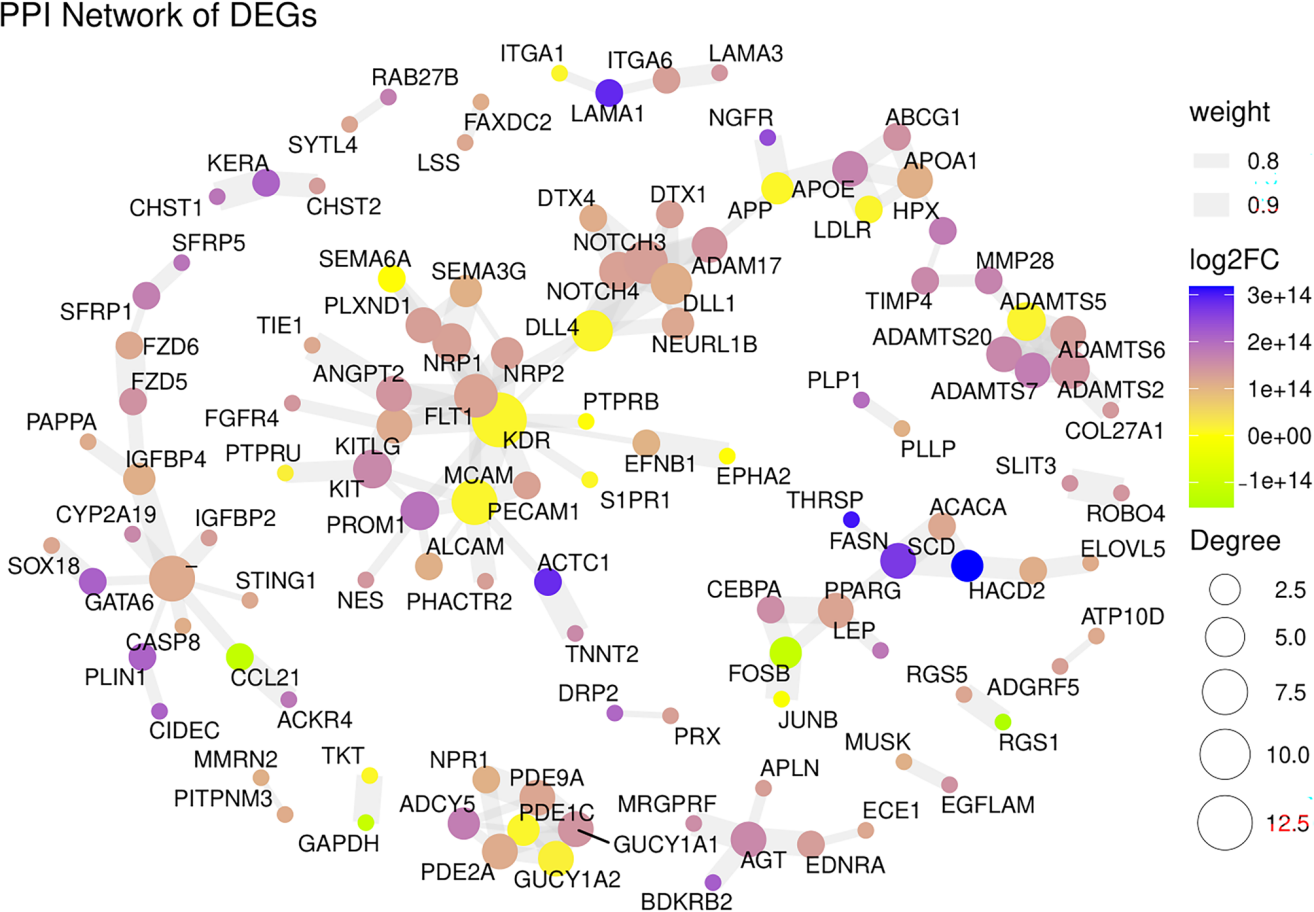
Protein–protein interaction (PPI) network of differentially expressed genes (DEGs) between high and low intramuscular fat (IMF) groups in Black Slavonian pigs. The network includes genes identified as significantly differentially expressed (adjusted *p* ≤ 0.05), with edges representing high-confidence interactions (STRING score ≥ 700). Node size corresponds to degree centrality (connectivity), and nodes are colour-coded by cluster membership, representing distinct functional modules identified using the Infomap community detection algorithm. Edge thickness is proportional to STRING interaction confidence. Only genes with successfully mapped Ensembl IDs and available cluster assignments are shown.

To enhance biological interpretation, functional modules within the network were identified using the Infomap clustering algorithm. A total of 26 distinct clusters were detected, representing putative functional sub-networks. These clusters highlight potential cooperative roles among genes involved in lipid biosynthesis, extracellular matrix remodelling, vascular development, and signal transduction. Notably, genes associated with lipid metabolism predominantly clustered together, supporting the functional coherence of DEGs in regulating IMF content. The visual representation of the network with cluster-based node colouring and interaction-weighted edges is shown in Fig. 8.

### Validation of RNA-seq results by RT-qPCR

To validate the reliability of the RNA-seq data, quantitative real-time PCR (qPCR) was performed on a subset of 10 differentially expressed genes randomly selected from top 30 DEGs: LIN7B, PHACTR2, CFH, GXYLT1, PLXND1, ECE1, FLNA, RFLNB, MYO1B, and NRG4 (Supplementary Information Figure S1a and S1b). The expression patterns detected by qPCR were highly consistent with the RNA-seq results. Specifically, eight of the ten genes showed statistically significant differential expression between the HIMF and LIMF groups in the same direction as observed by RNA-Seq. The remaining two genes, LIN7B and NRG4, also demonstrated concordant trends with the transcriptomic data, although the differences did not reach statistical significance (*p* > 0.05). Notably, NRG4 exhibited a near-significant expression difference (*p* = 0.0556), supporting its potential biological relevance. These findings confirm the results from the RNA-seq data and provide additional confidence in the observed differential expression profiles.

## Discussion

In this RNA-seq study, we uncovered significant transcriptomic differences in muscle tissue between Black Slavonian pigs with high vs. low IMF. The HIMF group showed upregulation of numerous genes involved in lipid metabolism and adipocyte differentiation, consistent with prior gene expression studies of porcine IMF^23–25^. Conversely, many genes related to muscle structure and oxidative metabolism were downregulated in high-IMF muscle tissue, suggesting a trade-off between adipogenesis and myogenesis. These results align with the well-documented notion that increased IMF is accompanied by activation of fat deposition pathways and reduction of certain muscle fibre pathways^26–28^. Overall, our findings confirm and extend the molecular framework of IMF regulation established in pigs through microarray and RNA-seq studies, while also highlighting breed-specific and novel candidate genes. To provide a comprehensive interpretation of the transcriptomic differences associated with intramuscular fat (IMF) content, we first focus on genes and pathways most significantly enriched. Specifically, we highlight three biologically relevant domains that emerged from our functional analyses: lipid metabolism and adipogenesis, extracellular matrix remodelling and vascular development, and Notch signaling linked to cell fate dynamics. These pathways form the backbone of our interpretation and are discussed in detail below, followed by transcriptome-wide insights from gene set enrichment analysis (GSEA), comparative integration with previous IMF-related studies, and a focused exploration of novel gene-level findings.

Gene Ontology analysis demonstrated enrichment of biological processes linked to fatty-acid transport, triglyceride biosynthesis, and adipocyte differentiation, with particularly strong activation of the peroxisome proliferator-activated receptor (PPAR) signalling pathway, as the main transcriptional regulator of adipogenesis^23,29^. Elevated expression of PLIN1, FABP4, CIDEC, and ADIPOQ, canonical PPAR targets, was observed in HIMF muscles, reflecting findings from previous high-IMF studies across several pig breeds^20,23,25,30,31^. CIDEC (also known as FSP27) encodes a lipid-droplet protein that promotes triglyceride storage by suppressing lipolysis^32^, while ADIPOQ and CEBPA are central to adipocyte maturation and lipid accumulation^33–35^. The confirmation of these genes across independent datasets emphasises the robustness of this adipogenic signatures as a determinant of marbling.

We also observed increased expression of LEP (leptin), a key adipokine secreted from intramuscular adipocytes. This agrees with earlier reports in Duroc and Laiwu pigs^23,36^ and supports the hypothesis that elevated IMF enhances endocrine cross-talk within muscle. Leptin signals via LEPR, whose genomic loci have been associated with IMF in GWAS^18^. Likewise, SCD (stearoyl-CoA desaturase) which encodes the enzyme converting saturated to monounsaturated fatty acids, was strongly upregulated in HIMF samples, consistent with its established role in determining IMF quality and fatty-acid composition^14,18,37–39^. As SCD activity drives oleate synthesis^40^, its elevated expression may directly enhance marbling quality in Black Slavonian pigs, which are characterised by superior sensory traits. This consistency across molecular and physiological evidence underscores the PPAR–SCD–ADIPOQ regulatory network as a central metabolic hub for intramuscular lipid deposition.

Beyond well-known adipogenic regulators, several genes not previously connected to IMF emerged as differentially expressed. For example, NPSR1 (neuropeptide S receptor)—one of the top upregulated genes—has recognised roles in neuro-immune signalling^41^ but an uncertain connection to adipogenesis. It might participate in muscle–nerve or inflammatory communication associated with adipocyte expansion. Similarly, transcription factor TFCP2L1 and other less-characterised DEGs may represent regulatory elements contributing indirectly to muscle tissue remodelling or metabolic adjustment. Such genes could reflect breed-specific transcriptional patterns in the Black Slavonian pig or the influence of distinct cellular compositions within marbled muscle, meriting future exploration through targeted GWAS or cell-type-resolved studies.

Our results also point to substantial ECM reorganisation accompanying IMF deposition. Numerous collagen and laminin genes, including COL1A1, COL5A1, and LAMA1, were upregulated, together with ECM-associated glycoproteins such as TNC. “ECM organisation” and “ECM–receptor interaction” were significantly enriched categories, aligning with previous reports that link connective-tissue dynamics to fatness^42^. ECM restructuring is a prerequisite for adipose expansion: adipocytes require a more compliant matrix to enlarge, as observed in human adipose tissue^43^. Therefore, enhanced collagen turnover and matrix deposition in HIMF muscles may provide the physical and biochemical environment necessary for lipid storage. Comparable phenomena have been reported in marbled cattle, where collagen networks support intramuscular adipose lobules^44^. The cross-species similarity suggests a conserved requirement for ECM plasticity during IMF development.

Parallel to ECM changes, our data revealed activation of angiogenic processes. Pro-angiogenic factors such as FGF2 were markedly upregulated in HIMF muscles, consistent with findings in Large White pigs^45^. Since adipocytes rely on oxygen and nutrient supply, increased capillary growth likely accompanies adipocyte proliferation. Upregulation of VEGFR1 and related vascular genes further supports this view, echoing results in beef cattle, where highly marbled muscles exhibit greater capillary density and angiogenic gene expression^46^. The coordination between adipogenesis and angiogenesis may be mediated by shared regulators, including PDGFRB—identified as a hub gene both in our dataset and in WGCNA analyses of IMF^47^. Pathways such as PI3K–Akt and MAPK, enriched here and in Dingyuan pigs^25^, further underline this integrated metabolic–vascular network. Collectively, these results emphasise that marbling reflects not only the activation of fat-storage genes but also the concurrent restructuring of muscle’s extracellular and vascular microenvironment.

Among DEGs, several components of the Notch pathway (NOTCH3, NOTCH4, DLL1, DLL4) were significantly upregulated. Notch signalling maintains muscle satellite-cell quiescence and regulates progenitor-cell differentiation^48^. Elevated Notch activity may restrict new fibre formation while encouraging alternative cell fates, including fibrogenic or endothelial lineages. Notch inhibition has been shown to enhance adipogenesis^49^, suggesting that the higher Notch expression observed in HIMF muscle might act as a fine-tuning mechanism balancing myogenic and adipogenic outcomes. Moreover, DLL4 and NOTCH4, typically expressed in endothelial cells, may reflect enhanced endothelial–Notch communication linked to angiogenesis. Similar associations were recently reported in single-cell studies, where MAML2, a Notch co-activator, was implicated in IMF regulation^34^. Altogether, our results propose that IMF accumulation involves Notch-mediated modulation of progenitor cell fate, coupling vascular growth and adipocyte differentiation.

While DEG-based analyses highlighted discrete biological categories, gene set enrichment analysis (GSEA) provided a systems-level overview. LIMF muscles exhibited enrichment in “Ribosome”, “Spliceosome”, and “RNA Polymerase” pathways (NES = 2.13, FDR < 0.001), suggesting elevated translational activity and energy turnover^50,51^. In contrast, HIMF muscles were enriched for “Regulation of lipolysis in adipocytes”, “ECM–receptor interaction”, and “Notch signalling” pathways^52–54^. The reciprocal enrichment patterns reflect coordinated shifts between anabolic muscle maintenance and lipid-storage programming, supporting the interpretation that IMF content arises from integrated metabolic and structural adaptation rather than isolated gene effects.

The transcriptomic patterns observed in Black Slavonian pigs show strong agreement with those reported in other breeds, underscoring conserved mechanisms of marbling. Upregulation of PPARG, FABP4, CIDEC, and ADIPOQ—well-known adipogenic markers—has been repeatedly documented in Duroc^23^, Large White, and Laiwu pigs, and these same genes were among the most strongly expressed in our HIMF samples. Processes such as fatty-acid oxidation, triglyceride synthesis, and PPAR-driven adipogenesis were common denominators across datasets^23^. This high concordance strengthens the evidence for these pathways as universal regulators of IMF and potential targets for breeding or nutritional modulation. Moreover, genes like SCD and LEP, widely acknowledged in Duroc and Iberian pigs, were also significantly upregulated in our dataset, reinforcing their cross-breed relevance^18,26^.

At the same time, our work provides new molecular insight into the Black Slavonian breed, which is characterised by its robust IMF and traditional extensive management. We identified several breed-specific candidates not highlighted elsewhere, including NPSR1 and TWIST2. The latter, a transcription factor that inhibits myogenesis and promotes adipose development in mice^55^, was moderately upregulated in our HIMF group, suggesting a possible role in pig marbling. Furthermore, protein-interaction network analysis identified hub genes such as YWHAH and HSP90AB1, involved in stress response and intracellular signalling^47^. These hubs may act as integrators connecting metabolic and structural pathways during IMF accretion.

Comparisons across studies reveal that while the major metabolic regulators are conserved, subtle transcriptomic differences emerge depending on genetic background, muscle type, and analytical approach. Zappaterra et al.^45^, for example, observed enrichment of cilia-related genes in Large White pigs, a feature absent in our dataset, whereas our results uniquely emphasise Notch and neuropeptide pathways. Similarly, some angiogenesis-related factors like NRP1/2, which were significant in our findings, have not been widely reported in other pig breeds. Such distinctions highlight the value of analysing diverse breeds, as they collectively build a more complete picture of IMF biology.

Beyond confirming previously known pathways, our dataset uncovers novel genes and processes not previously associated with IMF, expanding the current understanding of marbling at both molecular and cellular levels. Interestingly, many of these genes are not classical metabolic enzymes but rather structural or signalling molecules, indicating that muscle–fat interplay involves broader cellular systems than once assumed.

Among the most notable is EHD2 (EH-domain-containing 2), which was strongly upregulated in HIMF muscle. EHD2 stabilises caveolae—plasma-membrane invaginations rich in CAV1—and regulates lipid uptake and storage. Functional studies in mice demonstrate that EHD2 deletion impairs adipocyte lipid accumulation and suppresses PPARγ activity^56,57^. The concurrent upregulation of EHD2 and CAV1 in our HIMF samples suggests enhanced caveolar activity and lipid trafficking within muscle cells. This introduces a new dimension to marbling biology: the role of membrane architecture in determining lipid storage capacity. Importantly, increased caveolar stability may facilitate more efficient fatty acid uptake and intracellular lipid retention in intramuscular adipocytes, thereby supporting sustained triglyceride accumulation. Although direct functional validation in porcine muscle cells was beyond the scope of the present study, the observed expression pattern of EHD2 is biologically consistent with its established role in regulating lipid handling in adipose tissue.

Another interesting finding is the downregulation of NRG4 (neuregulin-4), a secreted growth factor known for its beneficial metabolic effects in brown adipose tissue^58^. Reduced NRG4 expression in high-IMF muscle may indicate altered paracrine communication between muscle and adipose cells, potentially contributing to distinct metabolic profiles of fatty versus lean muscles. To our knowledge, NRG4 has not been reported in pig IMF studies, reinforcing its novelty in this context. Given that NRG4 has been implicated in promoting lipid oxidation and metabolic homeostasis, its reduced expression in high-IMF muscle may reflect a shift towards lipid storage rather than energy utilisation. This finding suggests that intramuscular fat accumulation may involve not only enhanced adipogenic capacity but also suppression of signalling pathways that normally constrain excessive lipid deposition. Further functional studies will be required to clarify whether NRG4 acts directly within skeletal muscle or indirectly through muscle–adipose endocrine interactions.

Additionally, several cytoskeletal and adhesion genes were among the top DEGs, including UTRN, FLNA, DCHS1, and PTPRU, as well as ECM-associated genes HMCN1, LTBP2, EGFLAM, and F11R. Their consistent upregulation in HIMF muscles suggests structural reorganisation of muscle fibres and enhanced cell–matrix interactions as fat infiltrates the tissue. Increased expression of UTRN, a dystrophin paralogue, may help stabilise muscle membranes under mechanical stress imposed by expanding adipocytes, a phenomenon that has not been described in previous porcine studies. These structural and adhesion genes were not found in earlier RNA-seq reports on IMF, indicating a broader tissue remodelling component in Black Slavonian pigs.

Altogether, our results broaden the scope of genes and pathways implicated in IMF deposition. While reaffirming the central role of adipogenic regulators such as PPARG, SCD, and ADIPOQ, we highlight additional layers of regulation related to caveolar dynamics, ECM flexibility, and vascular–muscle crosstalk. These findings support a systems-level model in which marbling develops through the integration of metabolic, structural, and microenvironmental processes and suggest that breed-specific expression profiles can reveal new angles for genetic improvement in traditional pig breeds.

## Conclusion

This study provides novel insights into the molecular mechanisms underlying intramuscular fat (IMF) deposition in the Black Slavonian pig, a local breed known for high meat quality. RNA-sequencing revealed 457 differentially expressed genes between high- and low-IMF animals, including key regulators of lipid metabolism and adipogenesis such as SCD, FABP4, ADIPOQ, CIDEC, and PPARG. These genes, consistently highlighted in other pig breeds, reinforce their central role in IMF regulation and offer potential as biomarkers or targets in breeding programs.

Beyond well-established pathways, we identified genes linked to extracellular matrix remodeling, vascular function, and signaling (e.g., EHD2, NRG4, UTRN, FLNA and HMCN1), suggesting broader biological processes involved in marbling. These findings expand the current understanding of IMF biology and may reflect breed-specific mechanisms in the Black Slavonian pig.

Altogether, our results confirm the conserved nature of core adipogenic pathways while highlighting additional molecular features relevant to muscle fat deposition. This work contributes to the genomic characterization of an underrepresented breed and provides a foundation for future breeding and management strategies aimed at improving pork quality through enhanced IMF content.

## Methods

### Animals and experimental design

The animals in this study were bred on commercial farm and slaughtered in commercial slaughterhouse in Croatia, in accordance with national legislative and guidelines. The study was approved by the Faculty Bioethics Committee for research on animals in Osijek (602–04/19–01/04) and reported in compliance with ARRIVE guidelines (https://arriveguidelines.org). The study included 80 Black Slavonian pigs (42 castrated males and 38 gilts) in full-sib experimental design, with known pedigree information. In order to obtain experimental animals for this study, 16 sows were mated to 4 boars, and as a result, 80 piglets from 16 litters were selected for the trial and kept in two equally sized groups in two pens. From these 80 animals, 14 individuals with divergent intramuscular fat levels were selected as sib pairs for RNA-sequencing analysis, as described in more detail below. Piglets were weaned at ± 48 days of age, and all siblings were preserved in groups until slaughtering. Average live weight of piglets at weaning was 10.56 kg. Beside the regular ear tags, at the age of 5 days, each animal was marked subcutaneously with microchips, which were injected into the auricle base of the left ear using the disposable syringes provided with the microchips. In this way, using two systems for animal identification, we were able to precisely identify origin of the animal during the trial. Male piglets were castrated at age of 7 days. All animals were housed in controlled environment, in semi-intensive production system, where pigs had more than 2,5 m^2^ of closed space and also available access to outdoor space (concrete floor, without roof) of similar size. Pigs were fed with conventional diet and ad libitum regime as follows: commercial starter feed with 16% of crude protein after weaning until the piglets obtained average 25 kg of mass; finisher feed with 15% of crude protein until the average mass of 70 kg; finisher feed with 14% of crude protein until the end of trial. Pigs had free access to water throughout the entire experiment. Animals were slaughtered at approximate age of 315 days (10,5 months) and final weight of 141 kg. Slaughter was performed in four batches (approximately 20 animals per batch), and statistical testing confirmed that slaughter batch did not have a significant effect on IMF content. Prior to slaughter, the animals were fasted for 12 h. In the slaughterhouse, 10 min post slaughter, a sample of the *Longissimus thoracis et lumborum* (LTL) muscle was collected from each carcass and stored into liquid nitrogen container. After approximately 3 h post slaughter, samples were stored at − 80 °C until RNA isolation. For the purpose of intramuscular fat content determination, the LTL muscle sample was taken from the same place from each carcass 24 h post mortem. Intramuscular fat content was determined using Soxhlet extraction method (ISO 1443:1973) and quantified as the weight percentage of fresh muscle tissue sample. In addition, IMF was also detected using NIR^59^. After laboratory analysis of IMF determination for all 80 animals, pigs were categorised as the highest and lowest IMF within litter and gender.  Out of the 80 animals, 14 individuals were selected for transcriptomic analysis, forming 7 divergent sib-pairs, each comprising one animal with low IMF (LIMF) and one animal with high IMF (HIMF), representing the lowest and highest IMF quartiles, respectively.

### RNA preparation

The muscle tissue samples were delivered on dry-ice to the Novogene Sequencing Facility (Cambridge, United Kingdom) for RNA extraction. Total RNA was extracted using the TRIzol reagent (Invitrogen) according to the manufacturer’s recommendations. Quantity and integrity of isolated RNA was assessed using the RNA Nano 6000 Assay Kit of the Bioanalyzer 2100 system (Agilent Technologies, CA, USA). Based on this analysis, high quality of RNA was achieved according to RNA integrity number (RIN 7,8–8,6) and RNA concentration (84–235 ng/µL). After quality control procedures, the samples were sequenced using NovaSeq 6000 sequencing platform (Illumina Inc, CA, USA) and the paired-end 150 bp (PE150) strategy.

### Library preparation for transcriptome sequencing

The process began by extracting mRNA from total RNA using magnetic beads with poly-T oligo attachments. The extracted mRNA was then fragmented using a First Strand Synthesis Reaction Buffer (5X) and heat, while the first strand of cDNA was synthesized using a random hexamer primer and enzyme M-MuLV Reverse Transcriptase (RNase H-). This was followed by the synthesis of the second cDNA strand, involving DNA Polymerase I and RNase H to properly process the strand. The cDNA was then refined by modifying any overhanging ends into blunt ends through a combined action of exonuclease and polymerase.

Subsequently, the 3’ ends of the DNA fragments were adenylated, and adapters with a unique hairpin loop structure were attached, setting the stage for hybridization. To specifically select cDNA fragments within the range of 370 to 420 bp, the library fragments went through a purification process using the AMPure XP system (Beckman Coulter, Beverly, USA). PCR amplification was then performed on these fragments, employing Phusion High-Fidelity DNA polymerase, Universal PCR primers, and an Index Primer. The PCR products were purified using the AMPure XP system, and the quality of the resultant library was assessed using the Agilent Bioanalyzer 2100 system.

For the final steps, the index-coded samples were clustered using the cBot Cluster Generation System and the TruSeq PE Cluster Kit v3-cBot-HS from Illumina, strictly adhering to the manufacturer’s guidelines. The clustered library was then sequenced on an Illumina Novaseq platform, successfully generating paired-end reads of 150 bp each.

### Data quality control

The initial phase involved refining the raw fastq-format data using customized perl scripts. This crucial step aimed to enhance data quality by eliminating any reads that contained adapters, poly-N sequences, or low-quality reads. Consequently, we obtained ‘clean reads’. Concurrently, we quantified the Q20, Q30, and GC content of these clean reads to ensure their high quality. All subsequent analyses relied exclusively on this high-quality, clean data, providing a robust and accurate downstream processing.

### Reads mapping to the reference genome

The Hisat2 v2.0.5 tool^60^ was used to construct the index of the reference genome and to align the paired-end clean reads to the reference genome. We chose Hisat2 as the mapping tool because it can generate a database of splice junctions based on the gene model annotation file, which results in a better mapping outcome than other non-splice mapping tools.

### Novel transcripts prediction

For each sample, the aligned reads were assembled together using StringTie v1.3.3b^61^, employing a reference-based transcriptome assembly approach. StringTie is a sophisticated computational tool that utilizes a network flow algorithm, rooted in optimization theory, and pairs it with an optional de novo assembly component to construct transcripts from complex data sets. In comparative analyses involving both simulated and real data sets, StringTie demonstrates superior performance while delivering more comprehensive and precise gene reconstructions and offers enhanced accuracy in estimating expression levels.

### Quantification of gene expression level

The software featureCounts v1.5.0-p3^62^ was used to count the number of reads associated with each gene. Subsequently, the Fragments Per Kilobase of transcript per Million mapped reads (FPKM) value for each gene was computed, considering the gene’s length and the number of reads mapped to it.

### Differential expression analysis

The differential expression analysis between the two groups, based on high and low IMF content and comprising of seven biological replicates per each group, was conducted using the DESeq2 R package 1.20.0^63^. DESeq2 employs statistical methods to detect differential expression in digital gene expression data, utilizing a model predicated on the negative binomial distribution. P-values obtained from the analysis were then adjusted according to the Benjamini and Hochberg method, which is designed to control the false discovery rate (FDR). Genes that presented with an adjusted P-value of 0.05 or less were identified as being differentially expressed. Additionally, principal component analysis (PCA) of FPKM values was analysed in R (R Core Team^64^) for all samples, with aim to further evaluate group differences or possible sample duplications.

### Validation of RNA-Seq results by quantitative real-time PCR (q-PCR)

To validate the differential gene expression results obtained from RNA-Seq analysis, quantitative real-time PCR (q-PCR) was conducted on a subset of 10 randomly selected genes from the top 30 DEGs. The same RNA samples used for sequencing were employed to ensure consistency across assays. q-PCR was carried out using SYBR Green on a Applied Biosystems, StepOnePlus Real-Time PCR System, following the manufacturer’s protocol. qPCR reactions were carried out using gene-specific primers (Supplementary Information Table S6) under standard cycling conditions. Each differentially expressed group consisted of five independent biological replicates per gene. Relative gene expression was quantified using the comparative Ct (2⁻ΔΔCt) method, with ACTB as the reference gene which exhibited stable expression across all samples and no significant differential expression between experimental groups in the RNA-seq dataset. Statistical differences in qPCR expression between the HIMF and LIMF groups were assessed using the non-parametric Wilcoxon rank-sum test. Statistical significance was defined as adjusted *p* < 0.05. RT-qPCR results are presented as boxplots of relative expression values (2⁻ΔΔCt), with individual data points representing biological replicates. Significance levels are indicated as *p < 0.05, **p < 0.01, ***p < 0.001, and ****p < 0.0001.

#### GO and KEGG enrichment analysis of differentially expressed genes

Gene Ontology (GO) and Kyoto Encyclopedia of Genes and Genomes (KEGG) enrichment analyses were performed to gain insight into the biological functions and molecular pathways associated with differentially expressed genes. We used the clusterProfiler R package^65^, to conduct the enrichment analyses. This tool corrects for gene length bias and applies the Benjamini–Hochberg method to control the false discovery rate. For GO analysis, DEGs were annotated and categorized into three major domains: Biological Process (BP), Molecular Function (MF), and Cellular Component (CC). GO terms with adjusted *p*-values less than 0.05 were considered significantly enriched.

KEGG pathway analysis was performed to map DEGs to known signaling and metabolic pathways, allowing interpretation of their biological relevance in the context of IMF deposition. The analysis was also conducted in clusterProfiler, using the latest KEGG database for *Sus scrofa* as the organism reference. Significantly enriched KEGG pathways were identified using the same statistical threshold (adjusted *p*-value < 0.05).

To visualize and interpret the results, dot plots were generated for both GO terms and KEGG pathways, showing the top-ranked categories based on gene count and enrichment score. These functional annotations provided the basis for biological interpretation in the Results and Discussion sections, particularly highlighting the involvement of DEGs in lipid metabolism, angiogenesis, extracellular matrix remodeling, and cell adhesion pathways.

#### Gene set enrichment analysis

Both the GO and KEGG data sets were independently subjected to Gene Set Enrichment Analysis (GSEA) software^66^ using pre-ranked approach, providing a comprehensive insight into gene function and interactions in the context of phenotypes under study. GSEA is a computational method designed to assess whether an a priori set of genes exhibits statistically significant, concordant differences between the two phenotypes. All genes detected in the RNA-seq dataset (n = 32,125 genes) were included in the analysis and ranked according to their differential expression between the HIMF and LIMF groups using the gene-level ranking metric generated by the GSEA software. Gene sets were considered only if their size fell within the predefined limits (minimum 15 and maximum 5,000 genes), resulting in 961 gene sets for GO (454 enriched in HIMF group, and 507 enriched in LIMF group) and 315 for KEGG (225 enriched in HIMF group and 90 enriched in LIMF group) retained for analysis after filtering. Enrichment significance was assessed using nominal *p*-values and false discovery rate (FDR), with an FDR threshold of 25% applied in accordance with standard GSEA recommendations.

#### SNP, alternative splicing and protein–protein interactions analysis of differentially expressed genes

Genome Analysis Toolkit^67^ software was used to perform SNP calling. Raw VCF files were filtered using default parameters. As alternative splicing (AS) is an important mechanism in regulation of gene and protein expression, rMATS 4.1.0^68^ software was used to analyse AS events. Protein–protein interactions (PPI) analysis of differentially expressed genes was based on the STRING database^69^, in order to predict Protein–Protein Interactions.

## Supplementary Information

Supplementary Information 1.

Supplementary Information 2.

Supplementary Information 3.

Supplementary Information 4.

Supplementary Information 5.

Supplementary Information 6.

Supplementary Information 7.

## Data Availability

All data generated or analysed during this study are included in this published article (and its Supplementary Information files). Raw RNA sequencing data that we analysed in the current study are available from sequence read archive (SRA), NCBI BioProject under the accession number PRJNA1359133 (https://www.ncbi.nlm.nih.gov/sra/PRJNA1359133).
