# Supplementary figures and images for: Gene expression profiling by RNA-sequencing reveals regulators of intramuscular fat in Black Slavonian pigs
Source: Sci Rep. 2026 May 20;16:17856. doi: 10.1038/s41598-026-52510-x (PMC13249812; doi:10.1038/s41598-026-52510-x)

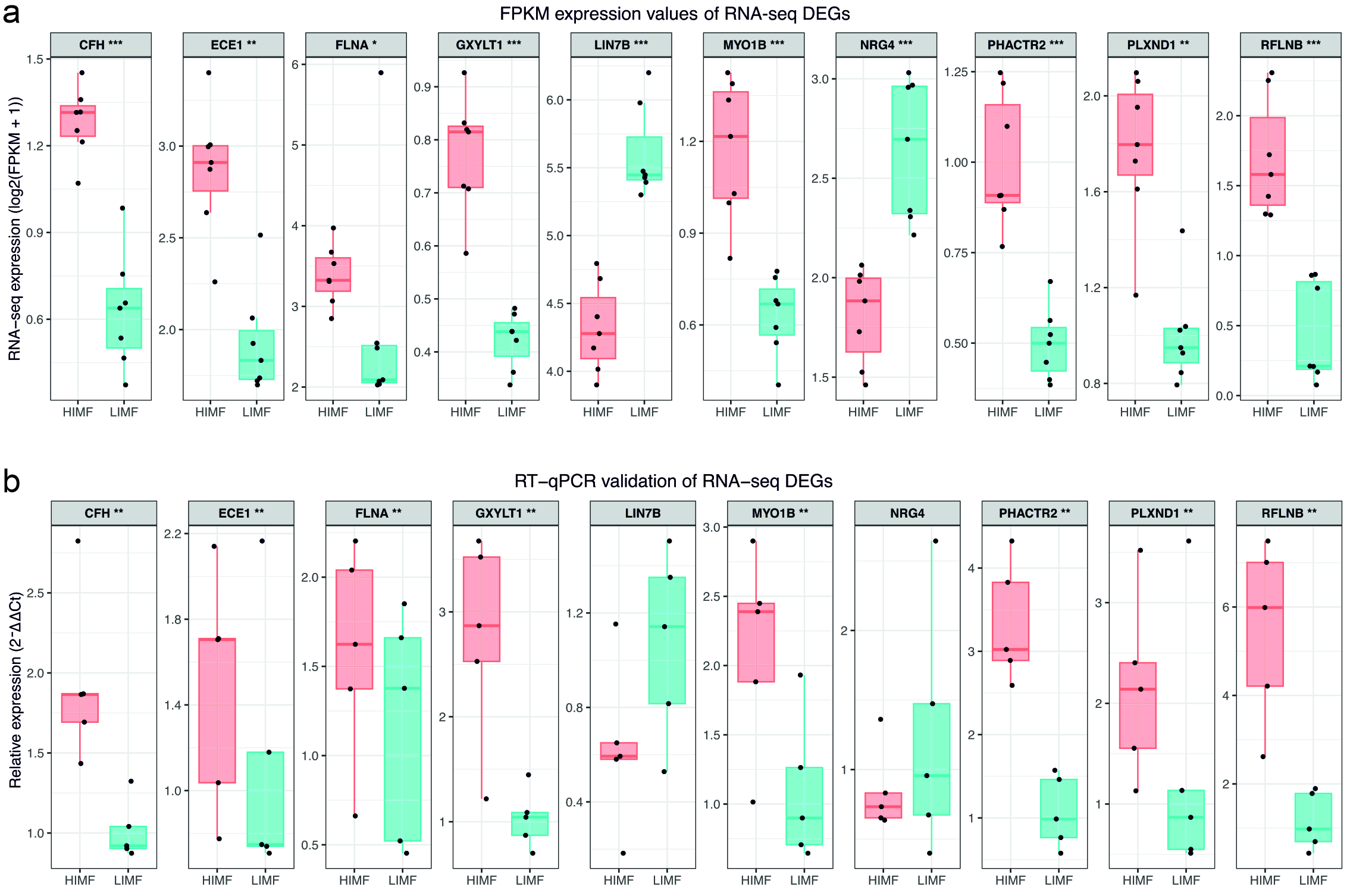

Supplement: Supplementary file 7 — Supplementary Information 7. [file 41598_2026_52510_MOESM7_ESM.tif]
